# Successful Renal Transplantation in a Patient With Senior-Loken Syndrome and Antiphospholipid Syndrome: A Case Report

**DOI:** 10.7759/cureus.45969

**Published:** 2023-09-26

**Authors:** Hamza Attiq, Ehsan Elahi, Muhammad Haseeb Ashraf, Hira Khalid

**Affiliations:** 1 Nephrology, Pakistan Kidney and Liver Institute and Research Centre, Lahore, PAK

**Keywords:** senior-loken syndrome, end-stage renal disease (esrd), nephronophthisis, antiphospholipid syndrome, renal transplant

## Abstract

Senior-Loken syndrome (SLS) is a rare autosomal recessive disorder affecting the eyes and the kidneys. It is an extremely rare disorder with an incidence of 1/1,000,000. Like most hereditary disorders, it is more commonly seen in families with consanguineous marriages.

Here, we present a case of a 35-year-old male with a complicated past medical history, who presented to us in the outpatient department for kidney transplant consideration. The patient was diagnosed case of Senior-Loken syndrome with a family history of autoimmune diseases, renal disease, and multiple unexplained miscarriages. He also had multiple dialysis access-related complications requiring frequent access changes. He previously had an unrelated pre-emptive renal transplant which resulted in graft failure within 48 hours. In view of his history, a prothrombotic condition was suspected and the patient was started on warfarin. Workup was positive for lupus anticoagulant and hematology recommended lifelong anticoagulation. The patient had a related renal transplant that was successful. He is now on apixaban and has not had any thrombotic complications to date.

This patient had antiphospholipid syndrome leading to multiple thrombotic events and a failed graft, but was never worked up for autoimmune disorders despite having a strong family history. His renal disease was presumed to be secondary to a rare condition - Senior-Loken syndrome and he was not investigated for a co-existing condition (e.g., antiphospholipid syndrome {APLS} in this case) which led to early graft failure. Hence when considering a patient for transplant, care should be taken to rule out autoimmune diseases and not ignore possible co-existing conditions in the presence of a renal pathology.

## Introduction

Senior-Loken syndrome (SLS) is a rare disease with patients having juvenile nephronophthisis progressing to end-stage renal disease and retinal degeneration progressing to blindness. SLS is a very rare autosomal recessive disorder with an incidence of 1/1,000,000. More incidence is seen in families with consanguineous marriages with very few cases reported worldwide [1].

Here, we describe a 35-year-old male who was on maintenance hemodialysis since July 2017 and presented to us for renal transplant evaluation. He was diagnosed case of Senior-Loken syndrome and had a past medical history significant for unrelated graft failure, arterio-venous fistula (AVF) thrombosis, and a family history significant for autoimmune diseases and recurrent miscarriages. His history was suggestive of a prothrombotic condition and he was diagnosed with antiphospholipid syndrome because of which he was started on warfarin. He later received a related kidney transplant and is currently taking apixaban. He has not had any thrombotic events post-transplant.

In this case, his renal disease was presumed to be secondary to a rare condition - Senior-Loken syndrome and he was not investigated for a coexisting condition despite his history being suggestive of one (e.g., APLS in this case) which led to early graft failure.

## Case presentation

This study is about a 35-year-old male who presented in the outpatient department for renal transplant evaluation. He was a known case of hypertension since 2006, CKD since 2008 (13 years back), and was diagnosed with vision disturbances in 2010 (record not available). His mother was a known case of SLE and his father a known case of ischemic heart disease, type 2 diabetes mellitus, and hypertension. His elder brother was diagnosed with kidney disease at the age of 12 years and was on maintenance hemodialysis. He later died in a car crash in 1990. He has two elder sisters, one of whom has a history of multiple miscarriages (×5) while the other has a history of pre-eclampsia. Two of his paternal cousins also had similar symptoms and were diagnosed with Senior-Loken syndrome in the United Kingdom. Also, he subsequently was diagnosed with SLS. Genetic testing could not be done due to the non-availability of facility.

**Figure 1:**
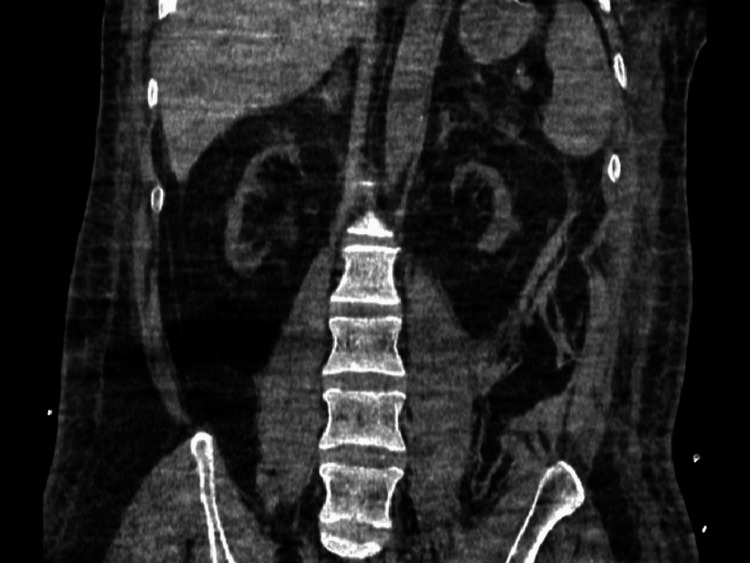
CT abdomen pelvis (coronal images) showing patient’s native end-stage kidneys.

His creatinine kept rising gradually and was 6 mg/dL in 2016 following which he had a pre-emptive live unrelated renal transplant done in 2017 which resulted in graft failure within 48 hours. Graft failure was secondary to thrombosis (as per the patient's narration, no record available). He was subsequently started on maintenance hemodialysis via temporary dialysis catheter. One month post-transplant, the graft had to be removed owing to persistent fever. He has since been on maintenance hemodialysis. His end-stage kidneys (at the time of presentation for the second renal transplant) are shown in Figure 1. He had a history of multiple dialysis catheter insertions. Three temporary catheters and three tunneled dialysis catheters were placed during this time. Catheters had to be frequently changed owing to no flow secondary to thrombi lodged in the lumens despite locking catheter lumens with unfractionated heparin. He also had two AV fistulas created on the left upper limb in 2017, which developed primary failure and could not be used. There was another AV fistula created on the right upper limb in 2019 which as per the history obtained did not work due to faulty needle insertion. Among his other comorbidities, he developed hyperthyroidism in 2011 and subsequently underwent radioactive iodine ablation to treat it. He has since been taking oral thyroxine. He also developed hepatitis C for which he also received treatment. He also had bilateral cataract surgery in 2018 and 2019.

His history was highly suggestive of a prothrombotic condition in light of his early graft failure, family history of autoimmune diseases, kidney disease and miscarriages, and multiple access failures. Hematology consultation was sought and a workup was advised for antiphospholipid syndrome (APLS), protein C and S deficiency, anti-thrombin 3 deficiency, factor V Leiden deficiency, and factor VIII elevation, which was later reviewed in the follow-up. Anticardiolipin IgG and IgM were negative, beta 2 glycoprotein 1 antibody IgG and IgM were negative but lupus anticoagulant returned positive. Protein C and S levels and factor VIII levels were normal. He was hence started on warfarin 5 mg once daily with close activated partial thromboplastin time (aPTT) monitoring. Hematology on the basis of a history of unprovoked thrombosis, repeated AVF closures, and positive lupus anticoagulant, recommended to put this patient on long-term anticoagulation. Hence warfarin was continued. In May 2021, he was admitted to the inpatient for renal transplantation surgery. Warfarin was stopped and unfractionated heparin was started. Heparin was continued in the intensive care unit after surgery. The hospitalization course was significant for abdominal re-exploration twice for perinephric hematomas. He became oligoanuric after the second perinephric hematoma development and was given three sessions of hemodialysis in the intensive care unit. Kidney function improved thereafter and the patient did not require further hemodialysis. Renal functions returned to normal in one week's time.

Post transplant the patient was initially given rivaroxaban which was then switched to apixaban on account of hematuria secondary to double J (DJ) stent placement. Post transplant he was never started on warfarin due to the patient’s apprehensions regarding frequent monitoring and risks of bleeding.

## Discussion

Senior-Loken syndrome (SLS) was first reported in 1961 by Loken et al., in a brother and sister duo, with the main features of nephronophthisis and Leber congenital amaurosis [2]. Senior-Loken syndrome is also known as renal retinal syndrome, juvenile nephronophthisis with Leber amaurosis, renal dysplasia, and retinal aplasia.

Renal nephronophthisis and retinal degeneration are common manifestations of SLS [3]. Renal nephronophthisis, also known as heterogeneous ciliary dysfunction or renal ciliopathy, is the most prevalent hereditary cause of chronic renal failure in the first two decades of life and results in cystic kidneys or renal cystic dysplasia. Infantile, juvenile, and adolescent types are the three clinical varieties, with the median age of onset being one year for infantile, 13 years for juvenile, and 19 years for adolescent types, depending on the age at which symptoms first appear and the genes that cause them. Genetic factors play a role in nephronophthisis (NPHP), and mutations in NPHP genes cause "ciliopathies," or changes in primary cilia, sensory organelles that connect mechanosensory, visual, osmotic, and other stimuli for cell cycle control. These changes affect multiple systems and cause a wide range of extra-renal manifestations [4,5]. About 14 different NPHP genes that are associated with nephronophthisis (NPHP) nephrocystin genes have been identified including (NPHP1, NPHP2, NPHP3, NPHP4, NPHP5, NPHP6, NPHP7, NPHP8 and NPHP9, NPHP10, NPHP11, NPHP12, NPHP13, and NPHPL1) [6-8].

The disease starts with polyuria and polydipsia and proceeds to end-stage renal disease with the later emergence of anemia and toxic accumulation of the blood products of protein breakdown (uremia). The corticomedullary conjunction exhibits enhanced echogenicity with renal cysts during radiological evaluation, such as ultrasonography. Interstitial fibrosis, tubular atrophy with corticomedullary cyst formation, and tubular basement membrane rupture are the salient features of the disease's histology [9].

Leber congenital amaurosis primarily affects the retina. While some individuals with Senior-Loken syndrome develop signs of Leber congenital amaurosis within the first few years of life, others do not experience vision issues until later in childhood. Retinitis pigmentosa, sector retinitis, Leber congenital amaurosis, and tapetoretinal degeneration are some of the retinal conditions related to SLS. The manifestation can be photophobia, nystagmus, hyperopia, night blindness, and drastically reduced visual fields, and vision can be limited to light perception [10].

Other symptoms including liver fibrosis or skeletal problems have been recorded in rare cases. There are a number of other syndromes associated with nephronophthisis and the findings such as “nephronophthisis, retinal dystrophy, severe deafness, diabetes mellitus, and obesity” constitute Alström syndrome; “retinal dystrophy, skeletal deformities, and respiratory insufficiency” are observed in Jeune's dystrophy; and in Bardet-Biedl syndrome, nephronophthisis may be present [11].

There are no proven remedies for nephronophthisis. Management of the disease primarily depends on delaying the progression of renal failure [12]. End-stage kidney disease requires dialysis or transplantation. After transplantation, kidney damage does not recur [13]. However, there is currently no treatment to prevent or stop the progression of vision loss due to retinal dystrophy, but various low-vision aids may be helpful for those who have remaining vision. Coexisting cataracts should be surgically corrected despite severe retinopathy as patients benefit from improved visual acuity following surgery [14]. Families and individuals with a family history of autoimmune disorders should be genetically screened for any of the NPHP genes.

Antiphospholipid syndrome is a multisystemic autoimmune disorder. It is characterized by the presence of a clinical criterion (thrombosis and/or miscarriages) combined with at least one positive laboratory criterion-Lupus anticoagulant, anticardiolipin antibody, and anti-beta 2-glycoprotein 1 antibody. Thromboprophylaxis is at the heart of management in patients with APLS. Patients with APLS need to be on lifelong anticoagulation with vitamin K antagonists (VKA) being the preferred choice due to the lower incidence of thrombotic events and bleeding incidence, especially in triple-positive patients [15]. Direct oral anticoagulants (DOACs) can be considered as an alternative to VKA in the case of contraindications to VKA, medical interactions with VKA, impracticable international normalized ratio (INR) measurement, problems finding the correct medication dose, or patient refusal for taking VKA [16]. Intensive patient education is required when using DOAC due to the short half-life of DOAC and adherence, persistence is essential. In case of the presence of any of the genes associated with APLS, the individuals should be referred for premarital genetic counseling, and consanguineous marriages should be discouraged [7].

## Conclusions

Patients with a strong medical history of AVF thrombosis and a family history of autoimmune diseases and miscarriages are at high risk of having an underlying autoimmune disease. However, our patient's renal disease was presumed to be secondary to a rare condition - Senior-Loken syndrome and he was not investigated for a coexisting condition (e.g., APLS in this case) which led to early graft failure. Hence when considering a patient for transplant, care should be taken to rule out autoimmune diseases and not ignore possible coexisting conditions in the presence of a renal pathology.
